# High-resolution analysis of copy number alterations and associated expression changes in ovarian tumors

**DOI:** 10.1186/1755-8794-2-21

**Published:** 2009-05-06

**Authors:** Peter M Haverty, Lawrence S Hon, Joshua S Kaminker, John Chant, Zemin Zhang

**Affiliations:** 1 Department of Bioinformatics, Genentech, Inc, South San Francisco, CA, USA; 2 Department of Molecular Biology, Genentech, Inc, South San Francisco, CA, USA

## Abstract

**Background:**

DNA copy number alterations are frequently observed in ovarian cancer, but it remains a challenge to identify the most relevant alterations and the specific causal genes in those regions.

**Methods:**

We obtained high-resolution 500K SNP array data for 52 ovarian tumors and identified the most statistically significant minimal genomic regions with the most prevalent and highest-level copy number alterations (recurrent CNAs). Within a region of recurrent CNA, comparison of expression levels in tumors with a given CNA to tumors lacking that CNA and to whole normal ovary samples was used to select genes with CNA-specific expression patterns. A public expression array data set of laser capture micro-dissected (LCM) non-malignant fallopian tube epithelia and LCM ovarian serous adenocarcinoma was used to evaluate the effect of cell-type mixture biases.

**Results:**

Fourteen recurrent deletions were detected on chromosomes 4, 6, 9, 12, 13, 15, 16, 17, 18, 22 and most prevalently on X and 8. Copy number and expression data suggest several apoptosis mediators as candidate drivers of the 8p deletions. Sixteen recurrent gains were identified on chromosomes 1, 2, 3, 5, 8, 10, 12, 15, 17, 19, and 20, with the most prevalent gains localized to 8q and 3q. Within the 8q amplicon, *PVT1*, but not *MYC*, was strongly over-expressed relative to tumors lacking this CNA and showed over-expression relative to normal ovary. Likewise, the cell polarity regulators *PRKCI *and *ECT2 *were identified as putative drivers of two distinct amplicons on 3q. Co-occurrence analyses suggested potential synergistic or antagonistic relationships between recurrent CNAs. Genes within regions of recurrent CNA showed an enrichment of Cancer Census genes, particularly when filtered for CNA-specific expression.

**Conclusion:**

These analyses provide detailed views of ovarian cancer genomic changes and highlight the benefits of using multiple reference sample types for the evaluation of CNA-specific expression changes.

## Background

Ovarian cancer is the fifth most common cancer among women and leading cause of death from gynecological cancers in the United States [1]. Treatment options include surgery, chemotherapy, and occasionally radiation therapy. To develop targeted therapies, which have the potential to be more effective and less toxic, candidate target genes must be identified. Trastuzumab, an antibody to HER2 for breast cancer therapy, provides a good example of an amplified cancer gene as a specific therapeutic target. As somatic DNA copy number alterations (CNAs) can indicate the presence of genes involved in tumorigenesis, studies of DNA instability in ovarian cancers could potentially lead to identification of causal genes and thus therapeutic targets. Indeed, several groups have used genomic technologies to systematically survey copy number changes in ovarian cancer [2-11].

Array comparative genomic hybridization (CGH) technology is frequently used for measuring copy number gains or losses. Early studies used Bacterial Artificial Chromosome arrays with resolutions of approximately 1 Mbp [2-4,6,8,9,11]. The Affymetrix 10K single nucleotide polymorphism (SNP) arrays, with the average resolution of 210 kb, have also been used to measure copy number changes in ovarian serous carcinoma [7,10]. Recently, the Affymetrix 500K SNP Chip has been applied to further survey the prevalence of copy number changes across the genome, focusing on rare micro-deletions [5]. Recurrent patterns of DNA amplification in regions including 3q26, 8q24, 12p13, and 20q13 were detected in multiple studies [3,5,6,9,11] (summarized in Additional file 1).

However, significant challenges remain in selecting the most important CNAs and in distinguishing driver genes from other passengers or bystanders. This is reminiscent of difficulties encountered in cancer genome re-sequencing projects where the discovery of driver somatic mutations is often complicated by the preponderance of passenger mutations [12-15]. Likewise, genes exhibiting copy number alterations are not necessarily functionally relevant in oncogenesis. In fact, due to the large size of most tumor amplicons, the vast majority of genes located in a given amplicon are likely passenger genes. The minimal region of overlap in an altered region can suggest a set of potential driver genes, but these regions can often span several megabase pairs. Currently, most studies have relied heavily on prior biological knowledge to select causal genes in amplified regions, which can potentially lead to inconsistency or domain-knowledge bias. For example, while the *PIK3CA *gene was selected from the 3q26 amplicon as the oncogene for subsequent functional analysis [16], the *PRKCI *gene from the same region was proposed by a different group to be the oncogene in ovarian cancer [17]. While many known or putative oncogenes were credited as the driver genes, some less characterized genes may have been overlooked. A systematic, unbiased approach should help find cancer-driving genes in ovarian cancer.

We have applied Affymetrix 500K SNP Chips to survey and compare copy number alterations (CNAs) in fresh-frozen ovarian tumor samples, of several subtypes, from 52 patients. Five additional samples were available from four patients allowing a comparison of CNAs from primary and metastatic samples as well as those in metastatic samples from different sites. To distinguish important changes from random CNAs, we have applied a statistical method (GISTIC: Genomic Identification of Significant Targets in Cancer) [18] that considers both the frequency and degree of copy number gains. This filtering leads to a relatively small set of genomic regions that reach statistical significance. To further evaluate the importance of genes in these regions to oncogenesis, we profiled the expression patterns of these tumors and whole ovary normal samples with Affymetrix U133A and B chips, filtering out genes with expression patterns that were inconsistent with their copy number. Combining copy number and expression data has been successful in other indications [19-21]. Lastly, as the ovarian surface epithelia (OSE) that are thought to give rise to the epithelial subtypes of ovarian cancer comprise a small percentage of the ovary [22], we also assessed the cancer-specific expression differences of genes in CNA regions using a public data set (GSE10971) consisting of Laser Capture Micro-dissected (LCM) normal fallopian epithelia and LCM serous adenocarcinoma.

## Methods

### Sample Information and SNP Array Analysis

Fifty-seven ovarian tumor samples from 52 patients were selected for analysis (Source: GeneLogic, Inc., Gaithersburg, MD). These tumors include 36 serous adenocarcinomas, 9 mullerian mixed tumors, 4 carcinomas of unspecified type, 3 clear cell carcinomas, 2 endometriod adenocarcinomas, 2 mucinous cystadenocarcinomas, and 1 granulosa cell tumor. All tumors were fresh frozen and possessed greater than 75% neoplastic cell content. Thirty-one samples were primary tumors while the remaining 26 were metastasized tumors. Forty-eight patients provided a single tumor. Matched primary and metastatic samples were available from three patients, one of which provided one primary and two metastatic samples. Two metastatic samples were available for one additional patient. Details regarding each sample, including tumor subtype, stage, and primary/metastasis status are available in Additional file 2.

For the Affymetrix 500K SNP array analysis genomic DNA preparation and chip processing were performed according to Affymetrix's recommended protocols. The array signal intensity CEL files were processed by dChip 2005 [23] (Build date Nov 30, 2005) using the PM/MM difference model and invariant set normalization. Data for 48 normal samples were downloaded from the Affymetrix website  and analyzed at the same time.

The dChip-normalized signal intensities were converted to log_2 _ratios and segmented as follows. For each autosomal probeset, the log_2 _tumor/normal ratio of each tumor sample was calculated using the average intensity for each probeset in the normal samples. For Chromosome X, the averages of the 20 female normal samples were used. The log_2 _ratios were centered to a median of zero and segmented using GLAD [24]. For each resulting genomic segment, GLAD estimates the mean, or inferred log_2 _ratio. DNA copy number was calculated as 2 ^(*inferred log ratio *+ 1)^. The raw and normalized SNP array data (CEL files and copy number) data are available at the NCBI GEO website (GSE11960). Hierarchical clustering of log_2 _ratio scale copy number values was performed with the Euclidean distance metric and complete linkage clustering.

### Comparison of CNAs to Germline Polymorphisms

A summary of 1193 published germline Copy Number Variants (CNVs) annotated as gain or loss was downloaded from version 5 of the Database of Genomic Variants [25].

### Prioritizing Altered Genomic Regions

Outliers in the segmented data were removed by merging segments of < 8 probesets with the neighboring segment having the most similar inferred log ratio. Probesets with an inferred log ratio > 0.3 or < -0.3 (~2.5 and ~1.6 copies) were classified as gain and loss, respectively. These values were selected to capture higher-confidence changes. Regions of significant gains or losses were identified using the GISTIC (Genomic Identification of Significant Targets in Cancer) application, version 0.9.2 [18].

GISTIC is similar in spirit to the STAC [26], CMAR [27] and MCR [28] methods in that it seeks to select the genomic regions containing the most important copy number alterations. GISTIC differs from these methods in that it assesses the statistical significance of the combination of alteration frequency and magnitude across the genome. In brief, GISTIC scores each probeset across the genome for the frequency with which it shows copy gain, times the average level of cancer/normal log_2 _ratio for the samples showing copy gain. The statistical significance of each score is then determined by comparison to the distribution of scores obtained from all permutations of the data. Multiple hypothesis testing is accounted for using the False Discovery Rate (FDR) Q-value statistic of Benjamini and Hochberg [29] and a cutoff of 0.25 (25% FDR) was used to select significant regions.

The GISTIC application selects the significant independent peaks on each chromosome by following a method called "Peel-Off". Peel-Off analyzes each peak on a chromosome in decreasing order of significance. For each peak it sets the log_2 _ratio of all probes in individual aberrations overlapping a more significant peak on the same chromosome to zero and then re-calculates the Q-value for the remaining peaks using the original permutation-derived null distribution G-scores. If a peak still meets the FDR cutoff of 0.25 after all more significant peaks on the chromosome have been peeled-off, it is reported in the final list of GISTIC peaks.

GISTIC determines the boundaries of each peak by serially removing one sample and repeating the analysis. The boundaries are taken to be the outer bounds of the start and end of the region across all iterations. Significant regions were assessed to be "broad" if the width of the GISTIC G-score peak at the level of significance (0.25 FDR) or half the maximal G-score for that peak spanned more than half the chromosome. Peaks narrower than this were judged to be "focal". DNA copy loss was analyzed in the same manner.

### Expression Microarray Analyses

All 57 samples were assayed for mRNA abundance using Affymetrix U133A and U133B microarrays. Affymetrix MAS 5.0 signal method [30] intensities were scaled to a mean of 500 (excluding the top and bottom 2% of values). For each gene, the probeset with the highest expression variance across cancer samples was selected unless noted otherwise. As a basis of comparison, 76 whole ovary samples from patients without ovarian cancer were also assayed in the same manner.

Copy number associated gene expression changes were assessed using a one-way ANOVA for each gene in each GISTIC peak. This ANOVA compared the average expression level of a given gene in three groups of samples: 1) cancer samples with the CNA in question, 2) cancer samples without the CNA in question, and 3) normal whole ovary samples from patients without ovarian cancer. The presence or absence of a CNA in a given sample was assessed using the same copy number cutoffs used for the GISTIC analysis. Following the fitting of the ANOVA, we applied Dunnett's Test to evaluate potential expression differences for the gene in question between groups 1 and 2 and between groups 1 and 3. Dunnett's test utilizes the within-groups variance from the ANOVA for the gene in question and accounts for the multiple tests for one gene. The resulting P-values for the ANOVA and two post-hoc comparisons were then separately corrected for multiple testing across genes using the Benjamini and Hochberg method [29]. Genes showing an FDR of < 5% for the ANOVA and either of the post-hoc tests were considered to have "CNA-specific expression". The ANOVA was performed using R and Dunnett's Test was performed using the "multcomp" package for R .

An external expression data set was downloaded from GEO [31] (GSE10971). Affymetrix U133 Plus 2.0 CEL files were processed using the MAS 5.0 method [30]. Intensities were scaled to a mean of 500 (excluding the top and bottom 2% of values). Twelve samples annotated as laser capture micro-dissected (LCM) non-malignant fallopian tube epithelium without *BRCA1 *or *BRCA2 *mutations were selected. Eleven LCM high-grade serous carcinoma samples were also selected after removing two additional samples from these same patients. Partek 6.4 was used to analyze cancer-specific gene expression differences [32]. Batch removal was applied to remove minor systematic differences between samples in this GEO data set that were run on different dates. This method applies a two-way ANOVA to each gene to estimate linear coefficients of expression variability due to sample batch and cancer/normal variables. The significance of cancer-specific expression differences was obtained from an F-test of the variability explained by the cancer/normal variable. The resulting P-values were corrected for multiple testing using the Benjamini and Hochberg method [29]. Significant expression differences were defined as a FDR < 1% and a cancer/normal ratio > 1.5 for gain or < 0.66 for loss. A gene was judged to show significant expression change if any probeset assigned to that gene was considered to be significant. When comparing this LCM data set to our own, we reduced potential difficulties related to microarray platform differences by analyzing the data sets separately and comparing them at the significant gene-list level, rather than comparing their raw data directly.

### Associations Among Prevalent Gains and Losses

The association between all pairs of the 30 genome regions identified by GISTIC was calculated using Pearson's correlation. P-values for each test were corrected for multiple testing using the Benjamini and Hochberg method [29]. Significant pairs were selected using a FDR cutoff of 0.25.

### LOH Analysis

Genotypes were generated using the BRLMM method (apt-probeset-genotype version 1.8.5 from the Affymetrix Power Tools package). The HMM-based method implemented in Partek 6.3b was used to detect LOH in each sample using the default parameters.

### Gene Set Analysis

The genes in the GISTIC peaks for gain and loss with CNA-specific expression patterns were analyzed for the relative abundance of the 364 genes listed in the Cancer Gene Census [33] (downloaded 2-6-2009). Entrez Gene IDs were used as the basis for matching genes and only genes represented by an Affymetrix U133A or U133B probeset were considered. Fisher's Exact Test was used to test for a positive association of Cancer Gene Census genes and genes located within a GISTIC peak, relative to all genes. Likewise Fisher's Exact Test was used to test for a positive association of Cancer Gene Census genes and genes located within a GISTIC peak showing a CNA-specific expression pattern.

### Pathway Analysis

Genes in the GISTIC peaks that showed CNA-specific expression were overlaid onto a global molecular network developed from information contained in the Ingenuity knowledge base (Ingenuity Systems^®^, ). Networks of these genes were then algorithmically generated based on their connectivity. Pathways Analysis also identified the pre-specified canonical pathways that were most over-represented in the data set. Fisher's Exact test was used to calculate a P-value for the association between the genes in the data set and the pathway. The resulting P-values were corrected for multiple testing using the Benjamini and Hochberg method [29].

### Gene Locations

SNP Chip probesets were mapped to the genome, NCBI assembly version 36, using annotation provided by the Affymetrix web site . Genes and Affymetrix expression probesets were localized on the genome by aligning RefSeq sequences and probeset targets to the genome, NCBI Version 36, using GMAP [34]. Cytoband and miRNA locations, for the NCBI Version 36 of the genome, were downloaded from the UCSC genome browser .

## Results

### General characteristics of copy number alterations in ovarian tumors

**Figure 1:**
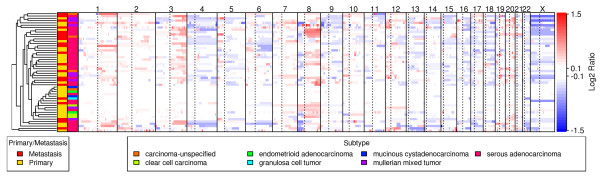
**Summary of Copy Number Changes for 52 Ovarian Tumors**. A heatmap and hierarchical clustering dendrogram of the inferred copy number values across each sample are depicted. In the heatmap, red represents copy gain and blue represents copy loss in units of log_2_(cancer/normal). Probes are proportionately spaced and arranged in genome order from 1 pter to Xqter. Vertical dashed lines represent the centromere locations. One sample is shown per patient. Color columns between the dendrogram and heatmap indicate sample subtypes and Primary/Metastasis status. Labels for these sample annotations are provided in a legend below the heatmap.

Data generated by profiling the 57 ovarian tumors were processed for inferred copy number using the dChip [23] and GLAD [24] methods. We first compared DNA copy number profiles of ovarian cancers of different subtypes from 52 distinct donors. While most of the samples are serous adenocarcinomas, there are a smaller number of tumors of other types, including clear cell carcinoma, endometrioid adenocarcinoma, granulosa cell tumor, and Mullerian mixed tumor (see Additional file 2). The most distinct cluster revealed by hierarchical clustering is made up of 15 samples that are predominantly primary tumors of subtypes that are neither mullerian mixed tumor nor serous adenocarcinoma (Fig. 1). These tumors are characterized by a dearth of CNAs, with the exception of gains on chromosome 8 in seven of these tumors. To control for any visual bias caused by the copy number heatmap, we compared the number of copy number breakpoints for each sample (See Additional file 3). Despite the apparent global differences in CNA across subtypes, the number and magnitude of gains and losses were not significantly different (P-values > 0.1). Given the low number of samples from the more rare subtypes and the limited tumor stage information for these samples, it is difficult to attribute the similarity of these samples to stage or subtype. Outside the cluster of 15 samples with infrequent CNAs, primary and metastatic samples were not distinguishable (Fig. 1).

### Samples from different sites in the same patient display similar CNA and expression profiles

To further evaluate the practicality of analyzing a mixed collection of primary and metastatic samples, we then examined whether different tumors from the same donors would exhibit similar CNAs. The inferred DNA copy numbers of 9 tumors from 4 distinct donors were examined using un-supervised hierarchical clustering analysis. It is apparent that although different donors display distinct patterns of DNA copy number gains and losses, tumors from different sites of the same donor show remarkable similarity and are clustered together (see Additional file 4). Such similarities apply to secondary tumors metastasized to different sites, and to primary and metastasized tumors in the same individual. These few samples also did not reveal any statistically significant genes, after multiple testing correction, in two-way ANOVAs testing for copy number or expression differences between primary and metastasized tumors controlling for patient ID. Therefore, despite the fact that the different ovarian tumors were collected from different sites, the genomic and expression patterns remain largely the same. Strong similarities in primary and metastatic CNA profiles have been reported before for ovarian cancer [35] and colorectal cancer [36]. For the subsequent analyses, we selected a single tumor sample to represent each distinct donor (see Additional file 2).

### Cancer-specific aberrations are quantitatively different than germline polymorphisms

In general, copy number changes we observed seem to have features distinct from the copy number variants (CNVs) recently reported to be present in the general population [25]. Notably, the copy number gains in ovarian cancer tend to be considerably broader than CNVs in the general population (P-value = 1.91e-94, two-sided t-test) (see Additional file 5). The median length of genomic regions with gain is 3.34 Mb in ovarian cancer, which is larger than the median length of 20.4 kb in the CNVs. Similarly, the regions of copy number loss in the ovarian cancer samples are also significantly larger than those of CNVs (see Additional file 5). More importantly, the distribution of tumor-associated copy number alterations in the genome is not uniform (Fig. 1), providing opportunity to infer oncogenic events based on the recurrent nature of such changes.

### Defining minimal tumor amplicons with statistical significance

Since numerous regions show some degree of copy number alteration, it is important to assign statistical significance to a given location. Selecting genome segments with a log_2 _ratio of < -0.3 and > 0.3 as "loss" and "gain", respectively, we then applied the GISTIC algorithm [18] to select the most statistically significant regions. For each probed location across the genome GISTIC calculates a "G-score" which is the fraction of samples showing gain at that probeset multiplied by the average log_2 _ratio for those samples showing gain. The significance of each G-score is assessed by comparison to a null-distribution of G-scores arising from permutations of the log_2 _ratios for all samples. The resulting P-value is then corrected for multiple testing across all probesets to generate a Q-value. Copy number loss is then analyzed in the same manner. The utility of this algorithm has been demonstrated in analyses of non-small cell lung cancer [37], glioblastoma [18], and breast tumors [38].

**Figure 2:**
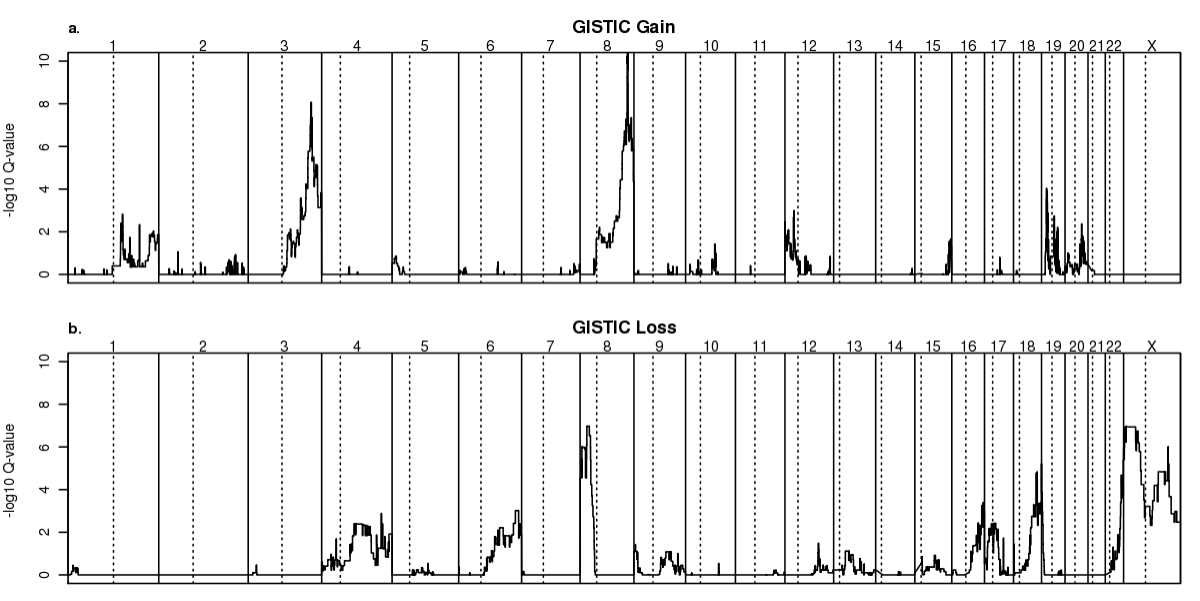
**Selection of Statistically Significant Recurrent Copy Number Alterations**. a) Genomic Identification of Significant Targets in Cancer (GISTIC) analysis of copy number gains was performed with 32 ovarian serous adenocarcinoma samples. GISTIC calculates the false discovery rate corrected significance (Q-value) of the frequency of gain and the average copy number for samples with gain at each probe position. Each point represents this Q-value for a SNP array probeset. Points are proportionately spaced and arranged in genome order from 1 pter to Xqter. Vertical dashed lines represent chromosome boundaries. b) GISTIC analysis of copy number losses, plotted as above. Gain and loss were specified as log_2_-transformed cancer/normal copy number ratios of > 0.3 and < -0.3, respectively.

Given the potential for the selection of different CNAs in different ovarian cancer subtypes (Fig. 1), we elected to focus our search for the most significant recurrent CNAs on the 32 serous adenocarcinoma samples. Despite the complexity of genomic profiles of individual tumors, the plot of GISTIC-based Q-values reveals a relatively simple pattern of amplification (Fig. 2a). Using an FDR cutoff of 25% as the threshold, we identified 16 distinct minimal regions ("Gistic Peaks") of gain on chromosomes 1, 2, 3, 5, 8, 10, 12, 15, 17, 19, and 20 (see Additional file 6). When expanded to the cytoband level, many of these regions can be matched to regions previously reported (see Additional file 1). GISTIC analysis of all 52 samples produced similar results with respect to the most significant gain and loss peaks.

### Prioritization of potential driver genes with CNA-specific gene expression

To further facilitate the enrichment of driver genes in each minimal amplicon, we compared the expression levels of genes within the bounds of each GISTIC peak among three groups of samples 1) tumors with the CNA in question 2) tumors without that CNA and 3) a pool of normal whole ovary samples. Genes showing significant expression differences among the three groups (1-way ANOVA) and between either groups 1 and 2 or 1 and 3 were judged to have significant "CNA-specific expression" (see Methods). While increased or decreased gene expression accompanying DNA gain or loss does not necessarily indicate functional significance, the lack of such a change would suggest that a gene is likely a passenger gene. Interestingly, only a small number of genes in the GISTIC peak regions display CNA-specific expression patterns (Additional files 6, 7, 8). By making these multiple comparisons we can avoid false-negatives that could arise due to other cancer-specific mechanisms of expression dis-regulation or differences in cell-type proportions in tumor and whole ovary normal samples.

The whole ovary normal samples available in our data set may not be optimal references. The epithelial portion of the ovary, which is believed to give rise to ovarian serous adenocaricinoma, comprises a small portion of the whole ovary [22]. Therefore, we have cross-referenced our expression findings to Laser Capture Micro-dissected (LCM) ovarian serous adenocarcinoma and LCM normal fallopian epithelia from a public data set (GSE10971), as described in the following sections.

### Enrichment for Cancer Census genes using copy number and gene expression

Although individual driver genes cannot be definitively determined in this study, we can estimate whether our approach leads to the enrichment of known cancer genes. All 364 genes in the Cancer Gene Census set [33] were selected to represent known cancer genes. We did not select only ovarian-cancer-specific genes as cancer genes tend to be relevant to more than one tissue. We detected a significant enrichment of Cancer Gene Census genes in the GISTIC Peak regions (one-sided Fisher's Exact, P-value = 4.06e-2, odds ratio 1.46). Selecting for only those genes in the GISTIC Peak regions with CNA-specific expression patterns showed an even higher degree of significance (one-sided Fisher's Exact Test, P-value = 1.08e-3, odds ratio 2.24).

### GISTIC gain peaks contain a small number of over-expressed genes

**Figure 3:**
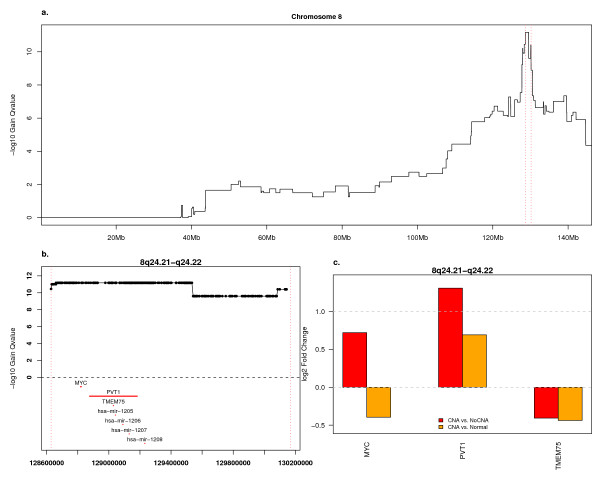
**Chromosome 8 Amplifications and Associated Expression Changes**. a) GISTIC Q-values for gain on chromosome 8, plotted as in Fig. 2. b) Close-up of the region of panel "a" indicated by vertical, red lines. The locations of all genes associated with a RefSeq transcript or Affymetrix probeset are indicated in red. c) Expression ratios for genes in the genome region depicted in panel "b" and represented on the U133A and B arrays. The probeset with the highest variance in cancer samples was selected for each gene. Red bars indicate the log_2 _ratio of the mean value in tumor samples with copy gain of this gene and the mean value in tumor samples without gain of this gene. Orange bars represent the log_2 _ratio of the mean value in samples with copy gain of this gene and the mean expression level in the normal whole ovary samples.

We examined several GISTIC peak regions in detail. The most significant region of gain was 8q24 where 59% of samples showed gain. This region contains only *MYC*, *PVT1*, *TMEM75 *and a cluster of four microRNAs (Fig. 3). In samples with this amplicon, *MYC*, *PVT1 *and *TMEM75 *do not show CNA-specific expression although the scores for *MYC *are suggestive (ANOVA FDR 0.02, Cancer with CNA vs. Cancer without CNA FDR 0.10, log_2 _ratio 0.75). Unexpectedly, in tumors with gain in this region, *MYC *is expressed at a level slightly less than the level in normal tissues (log_2 _ratio -0.12). Data on 25 of the 32 serous adenocarcinoma samples and 51 whole ovary normals from the Affymetrix U133 Plus 2.0 array shows similar data for *MYC*. However, this newer array offers a probeset with probes distributed along the length of *PVT1 *(1558290_a_at). This probeset shows that *PVT1 *is over-expressed relative to non-amplified samples (2.5×) and to normal (1.6×) (Fig. 3c). The LCM data did not show significant expression difference between cancer and normal samples for any gene at this locus.

**Figure 4:**
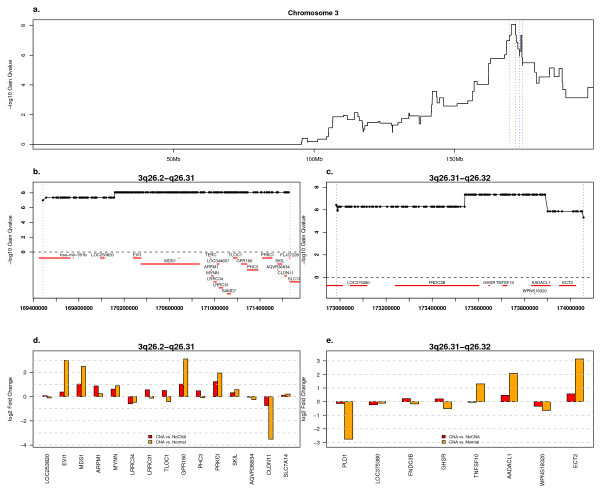
**Chromosome 3 Amplifications and Associated Expression Changes**. a) GISTIC *Q*-values for gain on chromosome 3, plotted as in Fig. 2. b) Close-up of the region of panel "a" indicated by vertical, red lines. The locations of all genes associated with a RefSeq transcript or Affymetrix probeset are indicated in red. c) Close-up of the region of panel "a" indicated by vertical, blue lines. The locations of all genes associated with a RefSeq transcript or an Affymetrix probeset are indicated in red. d) Expression ratios for genes in the genome region depicted in panel "b" and represented on the U133A and B arrays. The probeset with the highest variance in cancer samples was selected for each gene. Red bars indicate the log_2 _ratio of the mean value in tumor samples with copy gain of this gene and the mean value in tumor samples without gain of this gene. Orange bars represent the log_2 _ratio of the mean value in tumor samples with copy gain of this gene and the mean expression level in the normal samples. e) Expression ratios for genes in the genome region depicted in panel "c", plotted as in panel "d".

The region 3q26.2 is the next most significant. It shows gain slightly more often than 8q24 (63%), but at a lower level (2.91 copies among those showing gain relative to 3.21 copies, see Additional file 6). The most prominent peak on this chromosome is located at 3q26.2, but an interesting secondary peak can be observed at 3q26.31 (Fig. 4a). The first peak contains eight significantly over-expressed genes, *EVI1*, *MDS1*, *MYNN*, *TLOC1*, *GPR160*, *PHC3*, *PRKCI*, and *SKIL*. *PRKCI *has been shown to be a target of amplification in ovarian cancer that contributes to transformation in cooperation with mutant Ras in addition to contributing to anchorage independent growth [17]. These genes generally show dramatic up-regulation in samples with this amplicon, relative to normal ovary samples, and less over-expression in cancer samples with this amplicon relative to those without (Fig. 4d). Interestingly, *CLDN11 *shows dramatic under-expression in cancer, especially in samples with this amplicon. The LCM data set shows over-expression of *CLDN11*, *MYNN*, *PRKCI*, and *SKIL *in ovarian serous adenocarcinoma.

The 3q26.2 amplicon is adjacent to an interesting focal amplification. This region contains 5 genes, of which only *TNFSF10*, *AADACL1 *and *ECT2 *show over-expression in tumors with this amplification relative to normal whole ovary (Fig. 4e). A heatmap of aberrations on chromosome 3 (see Additional file 9) suggests that this adjacent peak results from one focal, high-level amplification in one sample in addition to frequent gain of a much wider region of 3q. This may explain why it not classified as a secondary significant peak by GISTIC's Peel-Off method (see Methods). Interestingly, *PIK3CA*, which has been suggested as an oncogene and driver of 3q gain in ovarian cancer [16], is not within either peak, although it is located in the shoulder region of the 3q26.2 peak. Gain of *PIK3CA *is less prevalent (49%) than at the major 3q peak in our samples (63%). The LCM data set shows significant over-expression of *ECT2 *(FDR 5.00e-4, cancer/normal ratio 1.88) and suggests over-expression of *TNFSF10 *(FDR 0.014, cancer/normal ratio 1.78).

**Figure 5:**
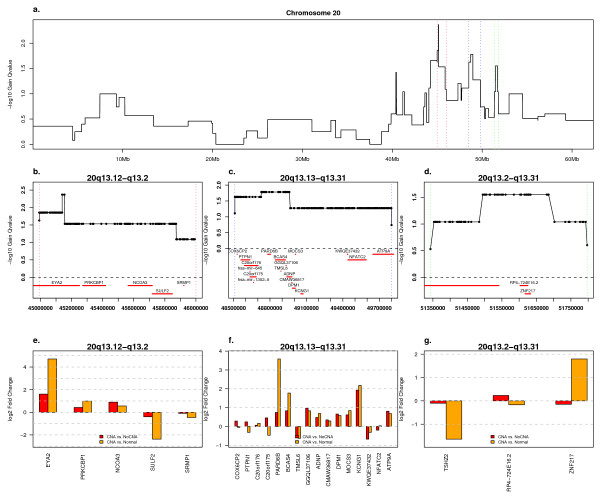
**Chromosome 20 Amplifications and Associated Expression Changes**. GISTIC Q-values and expression ratios for three regions of chromosome 20 plotted as in Fig. 2. Probeset 230533_at was eliminated as it mapped to the extreme 5' end of the *PRKCBP1 *transcript and disagreed with the majority of the other 6 probesets for that gene.

GISTIC selects one significant peak on chromosome 20 (see Additional file 6). The most significant peak, at 20q13.12, spans a genomic region that shows gain in 28% of samples and shows significant over-expression of *EYA2*, *PRKCBP1*, and *NCOA3 *(Fig. 5b, e). Closer inspection of the Q-values in this region reveals three narrow peaks (Fig. 5a), which is in line with previous findings of three cores of amplification in this region [39]. The second region, at 20q13.12-q13.13, shows gain in 31% of samples and spans 16 genes represented on the expression array (Fig. 5c, f). Of these *PARD6B*, *BCAS4*, and *KCNG1 *are expressed greater than two-fold higher in cancer samples with the amplification relative to normal (Fig. 5f). The third peak, also gained in 32% of samples, contains three genes represented on the expression array (Fig. 5d). Only *ZNF217*, a putative driver of 20q amplification in breast cancer [40] shows over-expression in cancer samples with this amplification (Fig. 5g). A broader and less prevalent (25%) peak containing *AURKA *is visible distal to this region. AURKA showed a log_2 _ratio of 0.95 in tumors with AURKA gain relative to those without and a log_2 _ratio of 1.43 in those tumors relative to normal whole ovary. Inspection of a heatmap of this region (See Additional file 9) shows some independent support for these secondary peaks although the focus of *ZNF217 *peak is formed by one narrow, high-level aberration. Of these chromosome 20 genes, only ZNF217 (FDR 2.67e-2, cancer/normal ratio 1.57) and AURKA (FDR 5.52e-5, cancer/normal ratio 2.32) were also significant in the LCM data set. Amplification of 20q13 may be particularly heterogeneous as *AURKA *[41], *TGIF2*, *PTPN1 *and *ZNF217 *[39], and *ADRM1 *[42], and other genes, have been cited as drivers of 20q13 amplification in ovarian cancer.

### Expression data points to potential tumor suppressors in broader recurrent deletions

GISTIC peaks for deletion were reported on chromosomes 4, 6, 8, 9, 12, 13, 15, 16, 17, 18, 19, 22, and X (see Additional file 7). The p-arms of chromosomes X and 8 are the dominant features (Fig. 2b), which we examined in further detail to identify potential tumor suppressors.

The GISTIC peak on chromosome X spans 4.6 Mb of Xp22.31 and includes 17 genes with RefSeq or Affymetrix annotation. Of these, 8 genes showed a significant decrease in expression in tumor samples with this deletion relative to tumors samples without this deletion: *ARSD*, *ARSE*, *MXRA5*, *PRKX*, *LOC729137*, *NLGN4X*, *HDHD1A*, and *STS *(see Additional files 7, 8).

**Figure 6:**
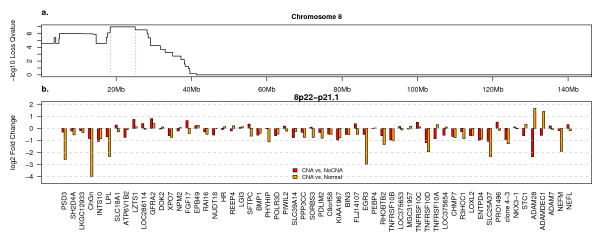
**Chromosome 8 Deletions and Associated Expression Changes**. a) GISTIC Q-values for loss on chromosome 8, plotted as in Fig. 2. b) Expression ratios for genes in the genome region depicted by blue dotted lines in panel "a" and represented on the HGU133A and B arrays. Red bars indicate the log_2 _ratio of the mean value in tumor samples with copy gain of this gene and the mean value in tumor samples without gain of this gene. Orange bars represent the log_2 _ratio of the mean value in tumor samples with copy gain of this gene and the mean expression level in the normal samples.

GISTIC identifies chromosome 8p as the autosome with the most statistically significant deletion. The most significant GISTIC peak in the 8p region spans 8p21.3 (Fig. 6). Although this extended peak contains 63 genes only 31 show significant down-regulation (see Additional files 7, 8). Among these are a number of pro-apoptotic proteins. The minimal peak includes the death receptors *TNFRSF10A *(*DR4*) and *TNFRSF10B *(*DR5*), the decoy death receptors *TNFRSF10C *and *D *(see Additional file 7) and all but *TNFRSF10C *show significant down-regulation. *EGR3*, which has been reported to induce the expression of the death ligand *FASL *[43] is among the most dramatically down-regulated genes in this GISTIC Peak.

Loss of Heterozygosity (LOH) was also analyzed to further characterize likely tumor suppressor genes (See Methods). LOH was indicated frequently throughout the genome, but without any clearly preferred regions (data not shown).

### Associations among prevalent gains and losses

The correlation of the occurrence of the recurrent CNAs was studied to assess potential interactions among the prevalent CNAs. The Pearson's correlation of the copy number values in all pairs of the 30 genomic regions identified by GISTIC (16 gain and 14 loss) was calculated. After correcting for multiple testing, 17 distinct pairs showed a FDR of < 25% (see Additional file 10).

Two adjacent GISTIC peaks on chromosome 19 are the most significantly correlated, likely because they are often co-amplified. The next most significant association is between 12p12.1, which over-expressed *BCAT1 *and is adjacent to *KRAS*, and 15q26.3, which contains *IGF1R *among other genes. Associations were detected between the *BCAT1 *and *IGF1R *peaks and the 8q24 peak containing *MYC *and *PVT1*. Curiously, the only anti-correlations among the significant interactions involve the *IGF1R *gain peak (4/5) or the *IGF2R *loss peak (1/5). These anti-correlations may indicate that alteration of signalling through the IGF axis may create selective pressure against acquiring certain other aberrations. Interestingly the deletion of the death receptors *TNFRSF10A *and *TNFRSF10B *on chromosome 8p did not correlate with the amplification of their ligand *TNFSF10 *on chromsome 3q.

## Discussion

We have applied high-resolution SNP arrays to survey the diversity of copy number alterations in ovarian cancer. Statistical analysis of the frequency and magnitude of these changes across the genome has identified the most significant changes. The analysis of expression changes correlated with these events has greatly reduced the field of potential "driver" genes for these most relevant CNAs. Co-occurrence analyses of these significant CNAs suggested potential synergistic or antagonistic relationships between some of the CNAs. Comparisons of subtypes and matched primary/metastatic samples have revealed potential differences among ovarian cancer subtypes and striking similarity among samples from the same patient. As a whole, these analyses provide a unique view of ovarian cancer and insight into key CNAs and their driver genes.

The strategy used here to evaluate CNA-specific expression changes highlights the difficulty of assigning CNA driver gene status on the basis of expression. Many likely candidate drivers were shown to have CNA-specific expression differences relative to tumors lacking the CNA in question and to whole ovary. However, the difference was often more dramatic when compared to normal samples than when compared to tumors lacking the CNA in question. Likely this is due to other mechanisms of expression dis-regulation in cancer that have an effect on expression similar to that of the CNA [28]. Additionally, differences between the mix of cell types in a tumor and whole ovary normal samples may cause certain disparities. For example, amplified *CLND11 *is reported as under-expressed when compared to our whole ovary normal samples, but is over-expressed when comparing LCM ovarian cancer and normal fallopian epithelium. Unexpected results such as this highlight the importance of using more than one reference expression level.

The expression pattern of the amplified *MYC *oncogene, especially relative to its neighbor PVT1, presents another interesting example of this phenomenon. The relatively minor expression change in tumors with *MYC *amplification may be due to over-expression of *MYC *in a subset of the non-amplified tumors through alternate mechanisms. The apparent under-expression of *MYC *upon amplification relative to whole ovary normal samples could be due to different cell type proportions in the tumor and normal samples. The LCM tumor and normal data set provides no clarity, as it did not show significant over-expression of *MYC *or *PVT1*.

Despite the long history of *MYC *as an oncogene, recent evidence suggests that *PVT1 *may be a driver of this amplicon. While *MYC *and *PVT1 *knock-downs both reduced proliferation of breast and ovarian cancer cell lines with amplification and over-expression of *MYC *and *PVT1*, knock-down of *PVT1 *in these lines also elicited a strong apoptotic response. Knock-down of *MYC *did not [44]. Mouse T-cell lymphomas show retroviral insertions at *PVT1 *and over-expression of *PVT1 *and one of *PVT1*'s microRNA products and more retroviral insertions are seen at the *PVT1 *locus than at the *MYC *locus [45]. Many transformed cell types, including neuroblastomas, express *PVT1 *and do not express *MYC *[46]. In the end, both genes may be important in the context of their amplification as *PVT1 *may be regulated by *MYC *[46] and *PVT1*-encoded microRNAs may regulate *MYC *[47].

The functions of these putative driver genes, selected on the basis of their location in the GISTIC peak and copy-driven expression, suggest an important role for apoptosis-related genes in this cohort of tumors. Genes showing significant CNA-specific expression changes were submitted to the Ingenuity Knowledge Base (Ingenuity Systems^®^, ) in order to determine if the GISTIC peaks highlight related genes. CNA-specific genes were selected rather than all genes with cancer-specific expression in order to detect direct relationships among genes in GISTIC peaks. The most significantly over-represented network inferred from pairs of associated genes documented in the Knowledge Base shows relationships among *CASP8*, *CASP10*, and *CFLAR *(gained on chromosome 2), *DIABLO *(gained on chromosome 12), *NFKBIB *(*IKB*) (gained on chromosome 19), and the *TNFRSF10 *family (deleted on chromosome 8) (see Additional file 11). Additionally, we have shown amplification of the *TFNSF10 *chromosome 3, although this was not among the GISTIC peaks. Analysis of the over-representation of genes from canonical pathways among the genes with significant CNA-specific expression changes shows that Death Receptor signaling is among the most significant pathways (Additional file 12). Taken as a whole, these findings indicate that modulation of apoptosis may be a major driving factor in the selection of CNAs.

## Conclusion

Based on these analyses, we believe that the identification of driver genes in tumor amplicons can be greatly facilitated by selecting statistically significant minimal recurrent amplicons and by studying gene expression patterns in conjunction with gene network data. The combination of the expression and high-resolution copy number data has provided a short list of candidate genes that are consistent with tumor driving roles.

## Competing interests

The authors declare that they have no competing interests.

## Authors' contributions

PMH carried out the copy number analyses, the analysis of gene expression in regions of CNA, and the pathway analyses, and drafted the manuscript. LSH participated in the "Cancer Gene" over-representation analysis. JSK participated in the gene network analysis. JC conceived of the study, and participated in its design and coordination. ZZ participated in study design and coordination and helped to draft the manuscript. All authors read and approved the final manuscript.

## Pre-publication history

The pre-publication history for this paper can be accessed here:



## Supplementary Material

Additional file 1 **Table S1: Comparison To Previously Reported Recurrent CNAs**. The table lists published examples of regions of gain and loss in ovarian cancer. Click here for file

Additional file 2 **Table S2: Sample Information**. The table provides the available clinical details for each tumor sample. Click here for file

Additional file 3 **Figure S1: Copy Number Alteration Trends by Subtype**. Raw copy number values were segmented into contiguous regions with the same copy number, or segments. The average log_2 _cancer/normal ratio of each segment is inferred as that segment's log_2 _ratio (See Methods). a) The number of transitions from segment to another (breakpoints) per sample, stratified by ovarian cancer subtype. b) The sum of segment log_2 _ratios that are > 0.3 (gain) in each sample, stratified by subtype. c) The sum of the log_2 _ratios for each segment that are < -0.3 (loss) in each sample, stratified by subtype. Click here for file

Additional file 4 **Figure S2: Copy Number Profiles of Matched Primary and Metastatic Samples**. Heatmap of nine samples from four patients plotted and clustered as in Fig. 1. The primary/metastasis status of each sample and the ID of patient from which each sample was derived are annotated by color columns between the dendrogram and heatmap. Labels for these sample annotations are provided in a legend below the heatmap. Click here for file

Additional file 5 **Figure S3: Comparison of CNA and CNV lengths**. The length of each genomic segment of contiguous copy number (see Methods) is plotted as a histogram. a) Lengths for segments with an average log_2 _ratio > 0.3 (gain) are shown in red. Lengths of polymorphisms in normal populations reported as a gain of copy number [25] are plotted in blue. b) Segments with an average log_2 _ratio < -0.3 (loss) are shown in red. Lengths of polymorphisms in normal populations reported as a loss of copy number [25] are plotted in blue. Click here for file

Additional file 6 **Table 1: Recurrent Gain Regions**. The table lists details regarding the significant regions of gain. Click here for file

Additional file 7 **Table 2: Recurrent Loss Regions**. The table lists details regarding the significant regions of loss. Click here for file

Additional file 8 **Table S3: Significant Expression Differences in CNAs**. The table provides statistics for the copy-number-associated expression changes in each region of gain or loss. Click here for file

Additional file 9 **Figure S4: GISTIC and Heatmaps for CNAs on Chromosomes 3, 8, and 20**. Details of the amplicon structure and statistical significance in 32 ovarian serous adenocarcinoma samples is presented for a) chromosome 3; b) chromosome 8; c) chromosome 20. Heatmaps and GISTIC amplification significance were prepared and plotted as in Figs.1 and 2. Click here for file

Additional file 10 **Table S4: Interactions Among CNAs**. The table provides statistics for the association among regions of gain and loss. Click here for file

Additional file 11 **Figure S5: Pathway Analysis of Genes with CNA-specific Expression**. Pathway analysis inferred connectivity among genes in GISTIC peak regions of gain and loss with CNA-specific expression. Network nodes have been colored using the log_2 _ratio for expression change in tumor samples with a given CNA relative to normal whole ovary. Green represents decreased expression and red indicates increased expression. White network nodes represent molecules not in our set of genes, but that are related to these genes through the Ingenuity database. Click here for file

Additional file 12 **Table S5: Canonical Pathways Enriched in Genes with CNA-specific Expression**. The table lists statistics regarding the over-representation of genes from canonical pathways in the regions of gain and loss. Click here for file
