# Supplementary figures and images for: High-resolution analysis of copy number alterations and associated expression changes in ovarian tumors
Source: BMC Med Genomics. 2009 May 6;2:21. doi: 10.1186/1755-8794-2-21 (PMC2694826; doi:10.1186/1755-8794-2-21)

### a. Breakpoints by Subtype

P-value:  $5.41e-01$

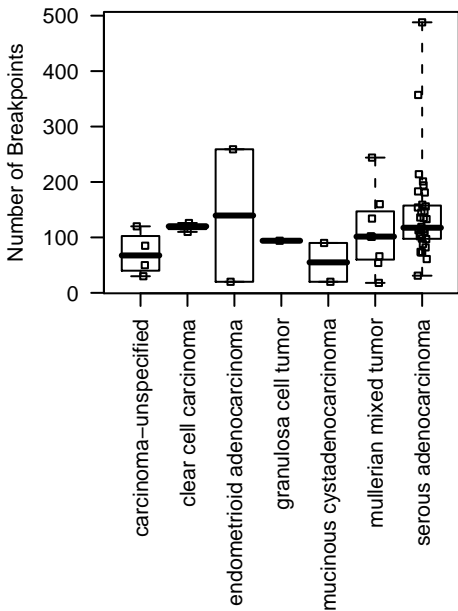

### b. Gains by Subtype

P-value:  $9.14e-01$

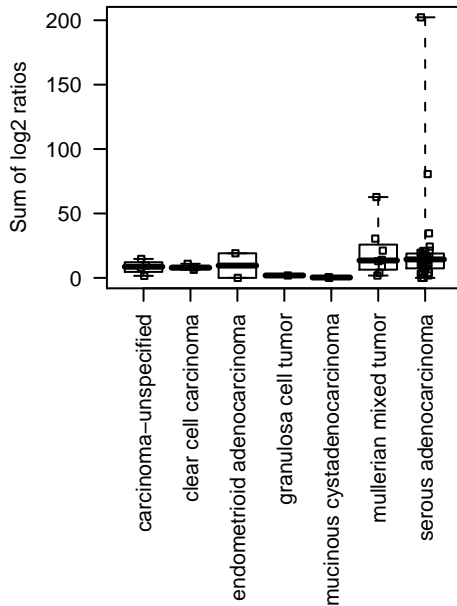

### c. Deletions by Subtype

P-value:  $1.06e-01$

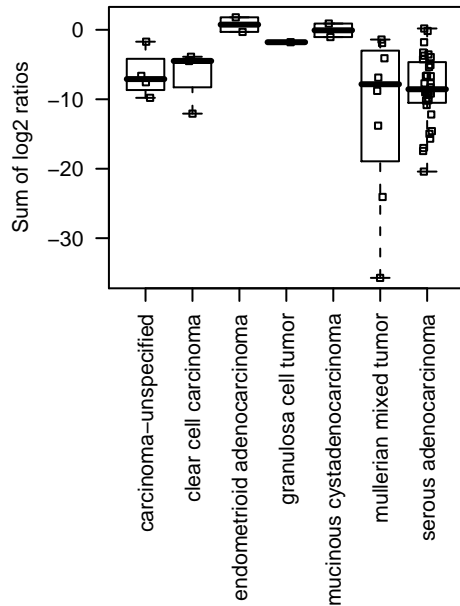

Supplement: Additional file 3 — Figure S1: Copy Number Alteration Trends by Subtype. Raw copy number values were segmented into contiguous regions with the same copy number, or segments. The average log2 cancer/normal ratio of each segment is inferred as that segment's log2 ratio (See Methods). a) The number of transitions from segment to another (breakpoints) per sample, stratified by ovarian cancer subtype. b) The sum of segment log2 ratios that are > 0.3 (gain) in each sample, stratified by subtype. c) The sum of the log2 ratios for each segment that are < -0.3 (loss) in each sample, stratified by subtype. [file 1755-8794-2-21-S3.pdf]

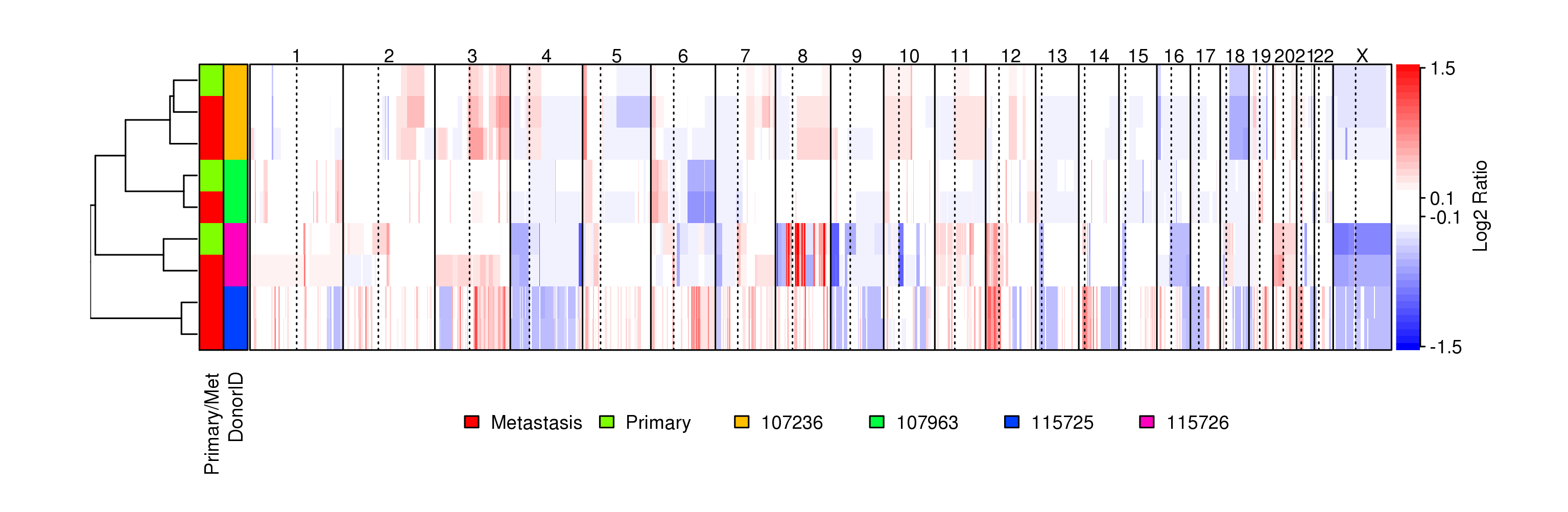

Supplement: Additional file 4 — Figure S2: Copy Number Profiles of Matched Primary and Metastatic Samples. Heatmap of nine samples from four patients plotted and clustered as in Fig. 1. The primary/metastasis status of each sample and the ID of patient from which each sample was derived are annotated by color columns between the dendrogram and heatmap. Labels for these sample annotations are provided in a legend below the heatmap. [file 1755-8794-2-21-S4.tiff]

### a. Copy Number Gains

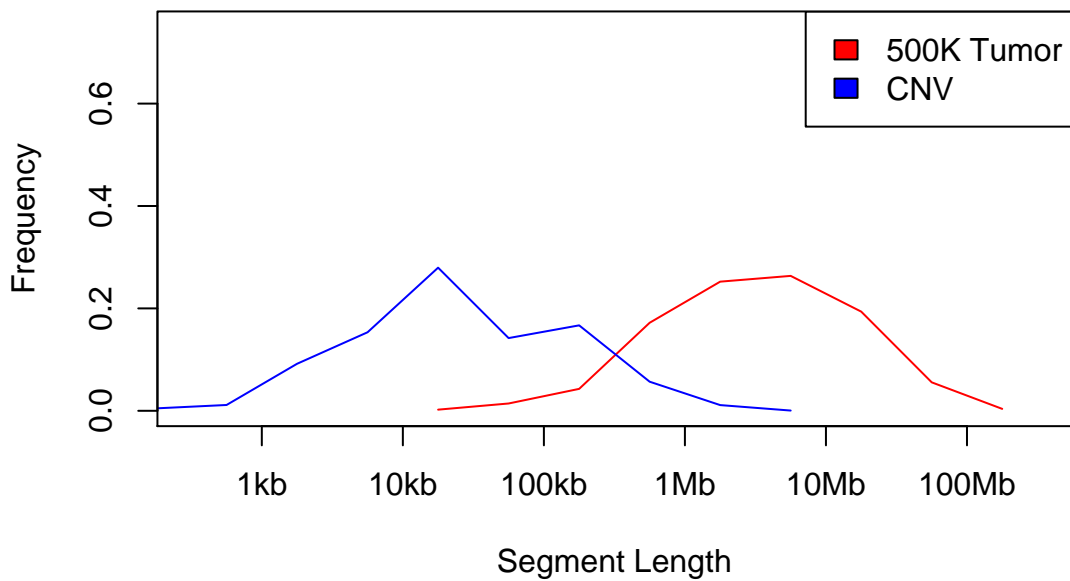

### b. Copy Number Losses

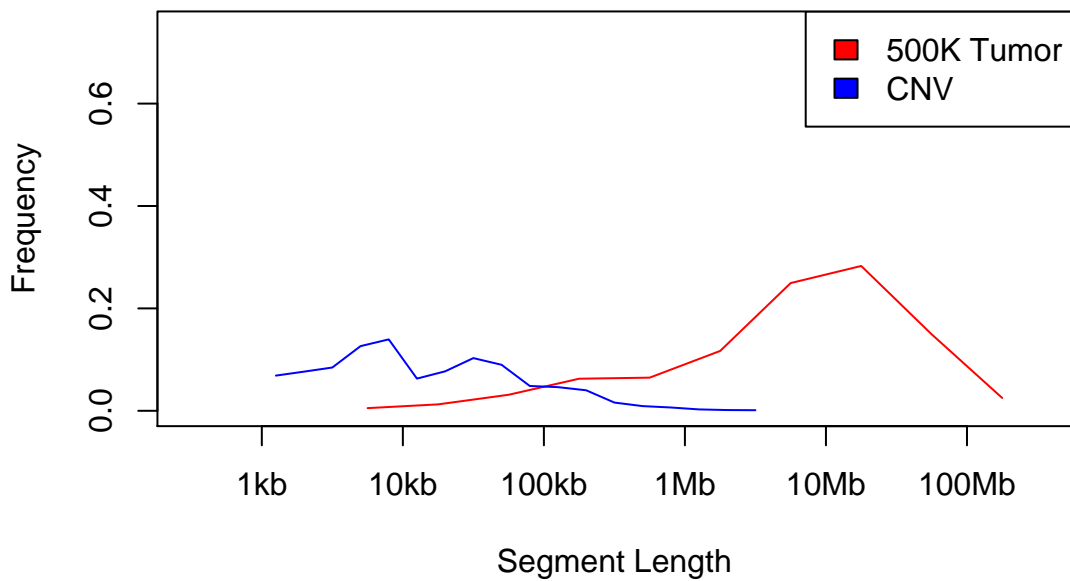

Supplement: Additional file 5 — Figure S3: Comparison of CNA and CNV lengths. The length of each genomic segment of contiguous copy number (see Methods) is plotted as a histogram. a) Lengths for segments with an average log2 ratio > 0.3 (gain) are shown in red. Lengths of polymorphisms in normal populations reported as a gain of copy number [25] are plotted in blue. b) Segments with an average log2 ratio < -0.3 (loss) are shown in red. Lengths of polymorphisms in normal populations reported as a loss of copy number [25] are plotted in blue. [file 1755-8794-2-21-S5.pdf]

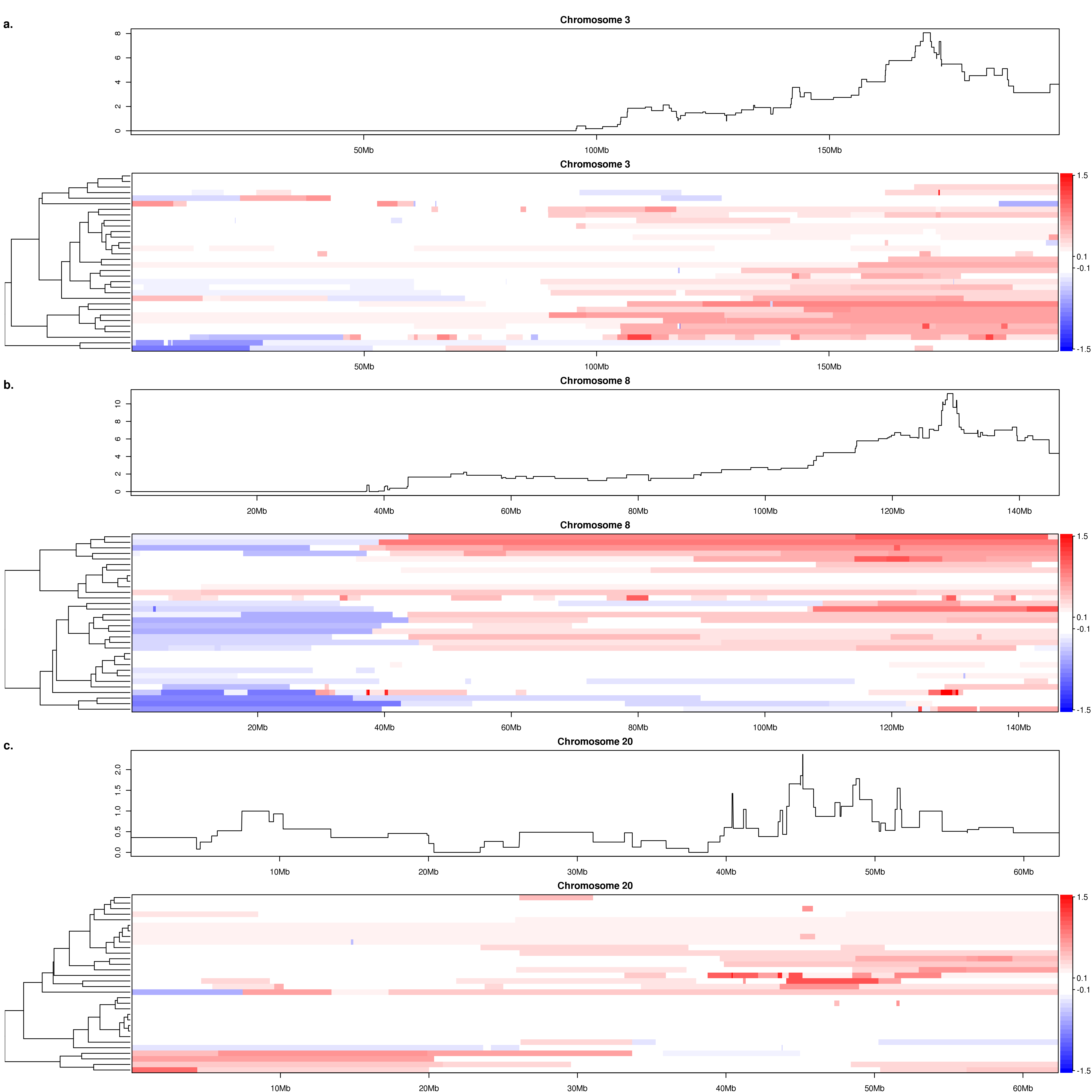

Supplement: Additional file 9 — Figure S4: GISTIC and Heatmaps for CNAs on Chromosomes 3, 8, and 20. Details of the amplicon structure and statistical significance in 32 ovarian serous adenocarcinoma samples is presented for a) chromosome 3; b) chromosome 8; c) chromosome 20. Heatmaps and GISTIC amplification significance were prepared and plotted as in Figs.1 and 2. [file 1755-8794-2-21-S9.tiff]

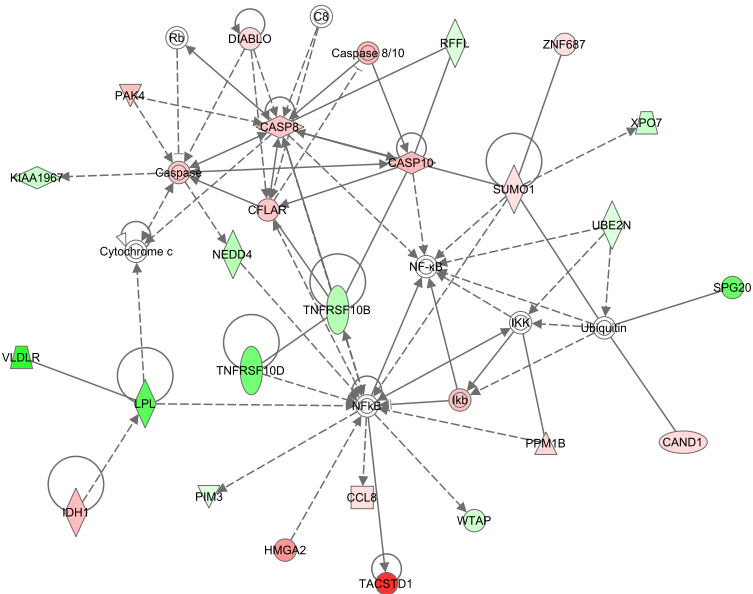

Supplement: Additional file 11 — Figure S5: Pathway Analysis of Genes with CNA-specific Expression. Pathway analysis inferred connectivity among genes in GISTIC peak regions of gain and loss with CNA-specific expression. Network nodes have been colored using the log2 ratio for expression change in tumor samples with a given CNA relative to normal whole ovary. Green represents decreased expression and red indicates increased expression. White network nodes represent molecules not in our set of genes, but that are related to these genes through the Ingenuity database. [file 1755-8794-2-21-S11.pdf]
